# The Georgia State Commission on Tuberculosis

**Published:** 1904-11

**Authors:** 


### EDITORIAL NOTES AND COMMENTS.

---

The Business office of THE JOURNAL-RECORD is No. 1007-1008 Century Building.

The Editorial office is 318-319-320 The Prudential.

Address all Business communications to Dr. M. B. Hutchins, Mgr.

Make remittances payable to THE ATLANTA JOURNAL-RECORD OF MEDICINE.

On matters pertaining to the Editorial and Original Communications address Dr. Bernard Wolff, Atlanta.

Reprints of original articles will be furnished at cost price. Requests for the same should always be made *on the manuscript.*

We will present, post-paid, on request, to each contributor of an original article, twenty (20) marked copies of THE JOURNAL-RECORD containing such article.

---

### THE GEORGIA STATE COMMISSION ON TUBERCULOSIS.

THE meeting of the State Commission on Tuberculosis at Macon, on Wednesday, October 19, represented the first organized movement ever made in the South for the protection of the people against their worst enemy in the form of disease.

It is a striking spectacle, that of physicians banded together for the purpose of guarding the public welfare, when the welfare of the public means their own financial detriment. It is a remarkable instance of the spirit of self-sacrifice which permeates the medical profession that they should cheerfully subordinate personal gain *pro bono publico.* Can such single-minded devotion to duty be paralleled outside of Fox's Book of Martyrs? The present Commission seeks to overthrow tuberculosis, not only without assistance of a material kind from the State, but in the face of general apathy on the part of the public. The work can not be furthered without funds, and where are these funds to come from? Out of the pockets of the members of the Commission.

The task set before the Commission to perform is herculean. The difficulty that hedges it about at the outset is lack of facility to gather statistical data, without which the work can not proceed. Tuberculosis is not a reportable disease as yet. Its chronicles may be found only in the case records of physicians, hospitals, clinics and in mortuary reports. These sources of information are often very incomplete and unsatisfactory, as well as difficult of access. Reliable data are thus difficult to secure, and in consequence the statistical report of the Commission must not be expected to be at all accurate. The means adopted by the Commission to obtain facts will be found outlined in the report of the Executive Committee of that body, which appears elsewhere in this journal. It is, briefly, that each district member of the Commission shall endeavor to secure the necessary information by appealing to every doctor of whatever school affiliation in his district to report to him the cases of tuberculosis which have come under his observation in the past year. Every medical reader of THE JOURNAL-RECORD in this State, whether or not he receive a letter from his district representative, is earnestly requested to send his report to the district member or to the secretary of the Commission. Blanks for this purpose will be furnished on application. Every man is expected to do his duty, and the duty is very simple. A postal card sent to the secretary or district member will bring the necessary blanks, and a few minutes' search of memory or memoranda will supply the desired information, which is to be entered on the blank and returned to the secretary. The work of the Commission will in this way be enormously assisted. There can be no doubt but that a desire for the success of the work of this Commission lies at the heart of every medical man in Georgia. It is a matter of State pride. Among all the States of the South, Georgia takes the leadership in this movement in the interest of progress and enlightenment. It is peculiarly an affair that concerns the medical profession of the State. It undertakes the self-allotted task without exoteric support except such as may be aroused and developed as the work proceeds and appeals to the public.

It is expected that each physician in the State will spread the propaganda by educating that part of the public which falls under his influence—educating it especially in the basic principles that consumption is an infectious disease, that it is a preventable disease, that it is a curable disease. If these facts are promulgated among the laity through sources that will carry conviction, the disease will be shorn of its awful strength—a strength bedded in ignorance and supported by weak human sympathy. Let the profession in the State get together, lay aside minor differences, and press on to the goal.

